# Underconfidence in peripheral vision

**DOI:** 10.1167/jov.21.6.2

**Published:** 2021-06-09

**Authors:** Matteo Toscani, Pascal Mamassian, Matteo Valsecchi

**Affiliations:** 1 Justus-Liebig-Universität, Gießen, Germany; 2 Laboratoire des systèmes perceptifs, Département d’études cognitives, École normale supérieure, PSL University, CNRS, Paris, France; 3 Bologna University, Bologna, Italy

**Keywords:** decision-making, metacognition, confidence, peripheral vision

## Abstract

Our visual experience appears uniform across the visual field, despite the poor resolution of peripheral vision. This may be because we do not notice that we are missing details in the periphery of our visual field and believe that peripheral vision is just as rich as central vision. In other words, the uniformity of the visual scene could be explained by a metacognitive bias. We deployed a confidence forced-choice method to measure metacognitive performance in peripheral as compared to central vision. Participants judged the orientation of gratings presented in central and peripheral vision, and reported whether they thought they were more likely to be correct in the perceptual decision for the central or for the peripheral stimulus. Observers were underconfident in the periphery: higher sensory evidence in the periphery was needed to equate confidence choices between central and peripheral perceptual decisions. When performance on the central and peripheral tasks was matched, observers were still more confident in their ability to report the orientation of the central gratings over the one of the peripheral gratings. In a second experiment, we measured metacognitive sensitivity, as the difference in perceptual sensitivity between perceptual decisions that are chosen with high confidence and decisions that are chosen with low confidence. Results showed that metacognitive sensitivity is lower when participants compare central to peripheral perceptual decisions compared to when they compare peripheral to peripheral or central to central perceptual decisions. In a third experiment, we showed that peripheral underconfidence does not arise because observers based confidence judgments on stimulus size or contrast range rather than on perceptual performance. Taken together, results indicate that humans are impaired in comparing central with peripheral perceptual performance, but metacognitive biases cannot explain our impression of uniformity, as this would require peripheral overconfidence.

## Introduction

The architecture of the visual system changes with eccentricity: the density of receptors and ganglion cells in the retina decreases with eccentricity and the corresponding cortical representations get smaller (Strasburger et al., 2011). This affects visual acuity, contrast sensitivity and color sensitivity (e.g., Hansen, Pracejus, & Gegenfurtner, 2009; Rovamo, Franssila, & Näsänen, 1992; Weale, 1953; Weymouth, 1958), and distorts the appearance of basic visual features like spatial frequency, luminance, chromatic saturation, or numerosity (e.g., Davis, 1990; Greenstein & Hood, 1981; McKeefry, Murray, & Parry, 2007; Valsecchi, Toscani, & Gegenfurtner, 2013). Peripheral vision has been characterized in controlled laboratory conditions, often by means of simple stimuli presented in isolation. However, in everyday life, the introspection of our vision across the visual field appears almost uniform: peripheral elements do not seem to be seriously blurred, achromatic, or dark.

To explain the mismatch between the processing limitations of peripheral vision and our subjective experience, different theories have been proposed. One possibility is that missing information is filled in with information from surrounding regions (i.e., filling-in mechanism). This can cause perception of brightness (e.g., Paradiso & Nakayama, 1991), color (e.g., Pinna, Brelstaff, & Spillmann, 2001), texture (Ramachandran & Gregory, 1991), or motion (e.g., Ramachandran, Gregory, & Aiken, 1991) at retinal locations where the corresponding sensory input is absent. Similar to the filling-in mechanism, we found that peripheral brightness appearance is “filled out” based on foveal information (Toscani, Gegenfurtner, & Valsecchi, 2017). Furthermore, when fixating for relatively long time a texture which presents a discontinuity between center and periphery, textures appear uniform (“uniformity illusion”), as if the centrally viewed pattern spreads to the periphery (Otten, Pinto, Paffen, Seth, & Kanai, 2017).

Neuroimaging and neurophysiological studies suggest that missing information is filled in at the earliest stages of cortical processing (e.g., Meng, Remus, & Tong, 2005; for a review, see Komatsu, 2006) consistent with the idea that the brain recreates the neural representations of visual information where it is absent or poor.

An alternative theory is that we do not fill in the missing information, we just are not aware that it is missing, as our conscious experience is built under the assumption that a complete visual picture of the observable world is present in our minds at every time (Dennett, 1991; O'Regan, 1992). Thus, rather than a perceptual explanation, the mismatch between conscious experience and peripheral vision could be the result of a metacognitive *inflation* bias (e.g., Odegaard, Chang, Lau, & Cheung, 2018; Solovey, Graney, & Lau, 2015).

Odegaard and colleagues (Odegaard et al., 2018) measured metacognitive performance in the periphery for crowded stimuli, as crowding presumably reflects the usual conditions of peripheral vision. Observers had to judge the orientation of a grating surrounded by two other gratings; after that, they had to indicate their confidence on their orientation judgment. On a control condition, the target stimulus was presented in isolation, thus being unaffected by crowding. Meta d-prime (Maniscalco & Lau, 2012) was used as a measure of metacognitive performance. Meta d-prime was lower in the crowded condition, indicating a metacognitive deficit.

Additionally, for all the trials where observers misjudged the grating's orientation, confidence ratings were higher in the crowded condition, indicating that the deficit was associated with a metacognitive bias: overconfidence for peripheral crowded stimulation. These results were interpreted as a sign of *inflation* for peripheral vision. Similar findings came from a detection task (Solovey, Graney, & Lau, 2015): participants adopted a conservative criterion at the center and liberal criterion at the periphery, i.e., they reported to have seen the target much more often at the periphery than at the center (more hits and false alarms in the periphery). Consistent with the presence of inflation in ecological conditions, while engaged in a driving simulation, participants showed liberal biases for unattended peripheral locations when detecting colors of pedestrians’ clothing (Li, Lau, & Odegaard, 2018).

However, in a study designed so that performance between perceptual judgments on crowded and un crowded peripheral stimuli was matched, observers were on average accurate, with no overconfidence bias (Barthelmé & Mamassian, 2010). Furthermore, the overconfidence bias found by Odegaard et al. (2018) may be specific to crowding, as peripheral vision in isolation was not tested against central vision.

Here we test metacognitive performance in peripheral as compared to central vision. To measure metacognition, we used a confidence forced-choice method (Barthelmé & Mamassian 2009, 2010; see for review: Mamassian, 2020), where participants report which one of two perceptual decisions they think they are more likely to have chosen correctly. This method is preferred to confidence rating also because confidence ratings may not actually reflect a metacognitive judgment but may prompt the observers to only judge the intensity of the sensorial stimulation according to multiple criteria along their internal sensory representation (e.g., Mamassian, 2016; Aitchison, Bang, Bahrami, & Latham, 2015). Ratings are also particularly vulnerable to between-participants noise, because different people can use the rating scale idiosyncratically (Morgan, Mason, & Solomon, 1997; for a review, see Mamassian, 2016), as well as to within-participants noise due to arousal fluctuations.

We measured metacognitive biases such as overconfidence or underconfidence by estimating the probability of higher confidence in the periphery after central and peripheral perceptual performance was matched. Results showed that observers were underconfident in the periphery: at matched perceptual performance, they were more likely to give higher confidence to the central judgment, or in other words, higher perceptual performance in periphery was needed to equate confidence choices between central and peripheral perceptual decisions.

Such biases can be explained with the assumption that rather than actually monitoring performance, confidence judgments are based on simple cues like perceived size or contrast (*cue-monitoring* hypothesis; Barthelmé & Mamassian, 2010). Although on average observers seem to follow performance rather than image cues (Barthelmé & Mamassian, 2010), there is some residual interindividual variability that can be accounted for by idiosyncratic and nonoptimal weighting of the different cues (de Gardelle & Mamassian, 2015). We ran an experiment to test whether underconfidence in the periphery can be explained by image properties such as size or contrast. Because of *cortical magnification* (for a review, see Strasburger, Rentschler, & Jüttner, 2011), stimuli in periphery need to be bigger than those presented centrally to equate perceptual performance, and likewise, stimuli in periphery need higher contrast than central ones. To anticipate our results, we showed that the peripheral underconfidence bias cannot be explained by an observer's strategy of using stimulus size or contrast as a proxy for confidence.

Metacognitive sensitivity was measured by estimating the increased perceptual sensitivity for high confidence perceptual decisions (de Gardelle & Mamassian, 2014; de Gardelle, Le Corre, & Mamassian, 2016). Metacognitive sensitivity is lower when observers compare the validity of their percept for central and peripheral locations than when they compared to central to central, or peripheral to peripheral. These results show that metacognition is specifically impaired when comparing perceptual performance at different eccentricities.

Since the metacognitive deficit we observed is associated with underconfidence (rather than overconfidence) in the periphery, it is inconsistent with the idea of peripheral *inflation* (Odegaard et al., 2018). Instead, we suggest that the richness of our experience of peripheral vision is due to filling in or filling out phenomena.

## Experiment 1

We investigated observers’ metacognitive performance and biases when they are comparing central and peripheral visual decisions.

### Methods

#### Observers

Fifteen students from the Justus-Liebig University of Giessen volunteered to take part in the experiment. All volunteers were naïve to the purpose of the experiment, and they had normal or corrected-to-normal visual acuity. Volunteers were reimbursed for their participation. They provided written informed consent in agreement with the Declaration of Helsinki, and all procedures were approved by the local ethics commission (approval number 2017-0030).

#### Apparatus

We used the psychtoolbox-3 software (Kleiner, Brainad, & Pelli, 2007) working on MATLAB (http://www.mathworks.com), to display the rendered movies on a linearized Eizo ColorEdge CG245W monitor (10 bits per color channel). Gaze position signals were recorded with a head-mounted, video-based eye tracker (EyeLink II; SR Research, Ottawa, ON), sampled at 500 Hz and monitored in real time. At the beginning of each experiment, the eye tracking system was calibrated, and the calibration was reexamined at the beginning of each trial.

#### Procedure

**Figure 1.**
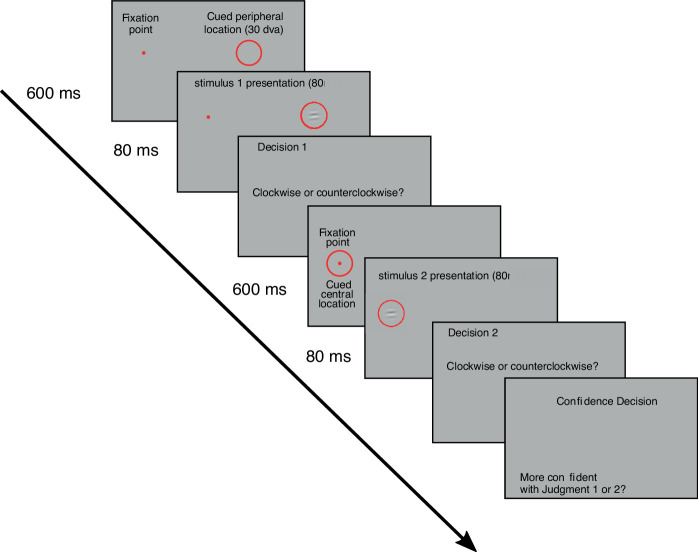
Experimental procedure. Each gray frame represents an example of the computer monitor as it was shown to the participants, with no black text, which is shown here for illustration purposes. The numbers on the left next to the time line (black continuous line) represent the presentation duration of each frame. When the time is not indicated, the participant was asked to provide a response with no time limit.

Participants sat 38 cm in front of the center of the computer screen with their head stabilized by a chinrest. The fixation point was presented together with a red circle indicating the position where a Gabor patch was going to be flashed (Figure 1). We determined whether the observers fixated at the fixation point by monitoring gaze position in real time. When they moved their gaze more than 3 dva (degrees of visual angle) away from the fixation point, observers were informed with a sound, and the current trial was aborted and repeated later. After 600 ms, an oriented Gabor was flashed for 80 ms at the cued location. The Gabor was rotated 5 degrees clockwise or counterclockwise at random, and observers had to report its orientation following a one-interval two-alternative forced choice procedure (1I-2AFC). This task was repeated in a second interval. In one interval the Gabor patch was flashed at the fixation point and in the other interval at 30 dva eccentricity on the right visual field. The presentation order was randomized every trial. The Gabor patch was arbitrarily chosen with a 5 dva standard deviation and 1 cycle/degree spatial frequency. After the two intervals, observers made a comparative judgment of their confidence about the correctness of their orientation decision (two-interval forced choice[2IFC]). The contrast of the Gabor patch was adaptively varied to keep performance between 50% and 95%, so that the confidence judgment would not be too obvious. To do so, at the end of each trial, we fitted a psychometric function to the orientation judgments for peripheral and central stimuli separately. Then we sampled performance from a uniform distribution [0.5, 0.95], and inverted the psychometric function to read out the contrast necessary to elicit the performance value we sampled. This was done for both intervals for the first 100 pairs. After that, we modeled the probability to respond higher confidence in the periphery as a function of the difference between peripheral and central performance with a cumulate Gaussian function. We used this model to sample a value of performance difference within the confidence judgment's dynamic range, i.e., sampling from a Gaussian function with the mean and standard deviations of the fitted cumulative Gaussian function. Then, we randomized performance for one of the two intervals (chosen at random) again within the [0.5, 0.95] range. Performance for the other interval was obtained by adding the sampled performance difference to the first interval's performance. Performance for the second interval was forced to not exceed perfect performance. The expected contrast to elicit the central and the peripheral performance was again read out from the fitted psychometric functions. Observers performed 500 pairs of orientation judgments and 500 confidence judgments. Most participants completed the experiment within two sessions of one hour. One participant needed three sessions and one four.

#### Analysis & results

We analyzed observers’ responses to assess metacognitive biases and performance when observers are asked to compare central and peripheral perceptual performance. Metacognitive biases are expressed as the ratio between the peripheral and the central sensory signals when confidence is equated (i.e., 50% chances for confidence chosen in the periphery). Values larger than one indicate underconfidence in the periphery, smaller values overconfidence. After establishing that observers could tell apart correct and incorrect perceptual decisions better than chance, both in the center and in the periphery, we numerically assessed metacognitive sensitivity.

##### Metacognitive biases

**Figure 2.**
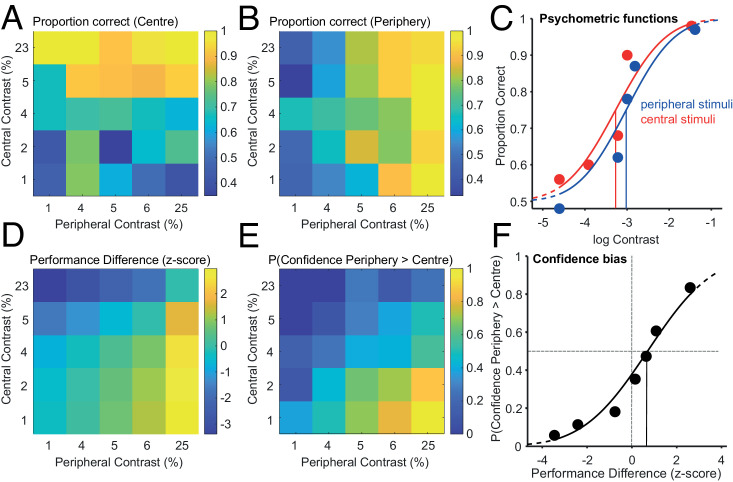
Metacognitive bias. A & B) Proportion of correct perceptual choices when the stimulus was presented in the center (A) and in the periphery (B). Peripheral contrast on the x-axis, central contrast on the y-axis, divided into five bins. The numbers on the axes correspond to the mean contrast of the stimuli assigned to the corresponding bin, expressed as a percentage. Proportion of correct responses is indicated by colormap with blue being low and yellow high proportions, as illustrated by the color bars on the right of each plot. C) Psychometric functions describing the proportion of correct perceptual choices as a function of the logarithm of contrast, for peripheral and central stimuli (blue and red, respectively). Discs represent the data points corresponding to A and B, and the lines are the fitted psychometric functions. Vertical lines indicate the estimated thresholds. D) Performance difference between peripheral and central stimuli. The difference is computed as the difference in z-scores of each performance and is indicated by the color bar on the right of the plot. The x- and y-axes are the same as for panels A and B. E) Probability of higher confidence in the periphery indicated by color bar on the right of the plot. The x- and y-axes are the same as for panels A and B. F) Probability of higher confidence in the periphery as a function of performance difference. Data from all trials were partitioned into seven bins, each one including the same number of trials. The continuous line represents the fitted cumulative Gaussian function f(x). The vertical continuous black line indicates the point of equal confidence (PEC) on the x-axis (i.e., performance difference for which the confidence level is 0.5).

To assess putative metacognitive biases, we estimated the performance difference between peripheral and central presentations for which observers had no confidence preference for either of these locations. To do so, we first partitioned the contrasts of the stimuli presented to each observer into five bins—with an equal number of trials per bin—for central and peripheral perceptual decisions separately, so that each pair of intervals was assigned to one cell of a 5 × 5 contrast matrix. By computing the relative frequency of correct response for each contrast cell of these matrices, we determined one performance matrix for the central and one for the peripheral orientation judgments (Figures 2A, B).

As expected, for the central perceptual decisions (Figure 2A), performance increased from the bottom of the plot to its top, indicating that performance increased with the central contrast independently of peripheral contrast. Likewise, for the peripheral perceptual decisions (Figure 2B), performance increased left to right, indicating that performance increased with peripheral contrast independently of central contrast. We fitted psychometric functions separately to the central and peripheral proportions of correct choices as a function of the log contrast (Figure 2C).

The next step in our analysis was to compare confidence choices for different levels of performance difference across peripheral and central presentations. For each trial, we read out from the psychometric functions the expected central and peripheral performance given the contrast of the central and peripheral stimuli. Central and peripheral expected performance were first z-transformed, so that their domain was changed from [0, 1] to [−∞,  ∞], then *performance* *difference* was computed by subtracting the transformed central expected performance from the peripheral one. As expected, large performance differences in favor of peripheral presentations are found when peripheral stimuli had a large contrast and central stimuli a low contrast (Figure 2D). For the same pairs of peripheral and central contrast, we then looked at the probability that the observer chose the peripheral decision with higher confidence. Similar to the performance difference analysis, we found high probabilities to choose the peripheral decision when peripheral stimuli had a large contrast and central stimuli a low contrast (Figure 2E). We could then directly compare confidence choices and performance difference (Figure 2F). The performance difference at which higher confidence was given with equal probability to the center or to the periphery (point of equal confidence [PEC]) was estimated by fitting a cumulative Gaussian to the relationship between the probability of higher confidence in the periphery and *performance* *difference.* The inverse of the fitted function at 0.5 is the *PEC*. If the PEC equals zero, observers are not biased in their confidence judgment, and they need the same perceptual performance for the center and periphery stimuli to be indifferent in their confidence judgment. A positive value indicates peripheral underconfidence, i.e., higher peripheral performance is needed to equate confidence in the center. Reversely, a negative value indicates peripheral overconfidence. On average, *PEC* was significantly larger than zero (mean z-score = 2.01; *t*(14) = 8.8074, *p* < 0.000001), indicating that lower performance was needed in the periphery to equate confidence probability.

##### Metacognitive sensitivity

The previous analysis was concerned with confidence bias and we now analyze the sensitivity with which observers were able to make their confidence judgments. We first determined whether observers could distinguish between correct and incorrect perceptual decisions better than chance. To do so, we fitted psychometric functions to the observers’ responses after splitting them based on their confidence choices (for a similar procedure, see de Gardelle & Mamassian, 2014; de Gardelle, Le Corre, & Mamassian 2016). Specifically, for each participant and separately for central and peripheral perceptual decisions, we separated the trials reported at higher confidence from the ones at lower confidence. In the central judgments, higher confidence is when observers choose higher confidence in the center, and consequently lower confidence is when they choose higher confidence in the periphery. The opposite relationship is true for the peripheral perceptual decisions. Then, we fitted cumulative Gaussian functions to these selections of trials to model the probability of correct perceptual decisions as a function of stimulus contrast expressed on a log scale. For the fitting procedure, we used the psignifit software (Schütt, Harmeling, Macke, & Wichmann, 2015). We read out the threshold parameter from the psychometric functions, indicating the contrast level at which the probability of correct answer was 75%, which we used as a measure of perceptual performance. Finally, we compared thresholds between high confidence and low confidence trials to assess whether confidence judgments were predictive of a difference in perceptual performance.

**Figure 3.**
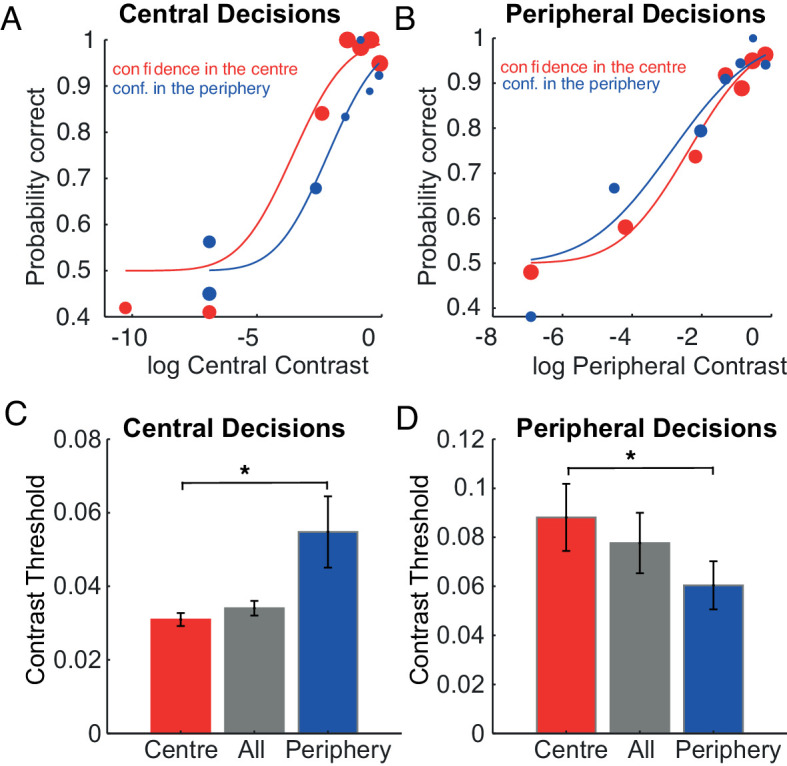
Metacognitive sensitivity. A & B) Perceptual performance for low and high confidence trials, for central (A) and peripheral (B) stimuli. Contrast is plotted on the x-axis, on a log scale. The blue data points represent the trials with higher confidence in the periphery (low confidence in A, high confidence in B), and red data points the trials higher confidence in the center (high confidence in A, low confidence in B). The size of the dots increases with the number of trials represented by each dot. The continuous lines represent the fitted psychometric functions, for the trials with high confidence in the center (red) and high confidence in the periphery (blue). C & D) Perceptual thresholds averaged across observers, for low and high confidence trials, for central (C) and peripheral (D) perceptual decisions. Thresholds, on the y-axis, are expressed in contrast units [0, 1]. The red bars represent the average threshold for the trials with confidence in the center (high confidence in C, low confidence in D), the blue bars represent the average threshold for the trials with confidence in the periphery (high confidence in D, low confidence in C). Gray bars represent the average threshold computed from the psychometric functions fitted to the whole response of each participant. Error bars represent the standard error of the mean. Horizontal black continuous line indicates which pairs of bars are significantly different from each other, with significance level (*) set to α = 0.0167, as determined by Bonferroni correction for three comparisons.

Figure 3A shows the psychometric functions for one participant for the central perceptual decisions, one for the trials judged with high confidence in the center (in red) and one for the trials with high confidence in the periphery (in blue). The former one is shifted to the left, indicating a lower threshold and thus higher perceptual performance for the trials judged with high confidence. In other words, when central perceptual decisions are judged with higher confidence, less contrast is needed to achieve the same performance than when they are judged with low confidence. The same is true for the peripheral perceptual decisions (Figure 3B): for the trials judged with high confidence (i.e., peripheral perceptual decisions with higher confidence in the periphery), less contrast is needed to achieve the same performance as for the low confidence trials (i.e., peripheral perceptual decisions with higher confidence in the center). This pattern of results is a signature of metacognitive ability (de Gardelle & Mamassian, 2014; de Gardelle, Le Corre, & Mamassian 2016).

Figure 3C shows the thresholds averaged across observers for the central perceptual decisions. Paired *t*-tests indicate that thresholds are lower for the high confidence trials, i.e., for the ones with high confidence response in the center (red bar), than for the low confidence trials (blue bar) (*t*(14) = 2.733, *p* = 0.016). On average, the threshold from the psychometric functions computed on all the trials (gray bars) is in between the one computed for the high confidence and the one for the low confidence trials. Likewise, Figure 3D shows the thresholds averaged across observers for the peripheral perceptual decisions. Paired *t*-tests indicate that thresholds are lower for the high confidence trials, i.e., for the ones with high confidence response in the periphery (blue bar) than for the low confidence trials (red bar) (*t*(14) = 7.7, *p* < 0.00001).

This result indicates that confidence judgments made by observers have access to some knowledge about their sensory uncertainty (metaperceptual sensitivity). To quantify metaperceptual sensitivity, we used the thresholds to compute a *Confidence Modulation Index* (*CMI*) (de Gardelle & Mamassian, 2014; de Gardelle, Le Corre, & Mamassian 2016). We started by computing sensory sensitivity as the inverse of the threshold (i.e., 1/σ). *CMI* was defined to describe changes in sensitivity between low (1/σ_*low*_) and high (1/σ_*high*_) confidence trials

(1) $$CMI=100\;\frac{\frac{1}{\sigma_{low}}\;-\;\frac{1}{\sigma_{high}}}{\frac{1}{2}\left(\frac{1}{\sigma_{low}}\;+\;\frac{1}{\sigma_{high}}\right)}$$

with σ_*low*_ and σ_*high*_ being the thresholds computed by fitting psychometric functions and converted into linear contrast units. The *CMI* indicates the relative sensitivity gain for high as compared to low confidence trials as a unit-free measure. We computed this index separately for central and peripheral perceptual choices. *CMI* is on average 39.1% and 39% for central and peripheral choices, respectively. *T*-tests across participants indicate that CMIs were positive in both cases (*t*(14) = 2.8, *p* < 0.05; *t*(14) = 7.87, *p* < 0.05; for central and peripheral choices, respectively).

The motivation of our study was to investigate metacognitive abilities in comparing our central and peripheral perception. We found that observers were able to monitor their perceptual performance in both central and peripheral vision (confidence modulation indices close to 39%) but they were underconfident for the peripheral decision as compared to the central one (point of equal confidence close to 2.01).

Previous results (Odegaard et al., 2018) suggest that peripheral vision is characterized by poor metacognitive performance. If we try to explain perceptual uniformity, however, the comparison which is most relevant is the one between central and peripheral vision. With a second experiment, we aimed at determining whether metacognitive performance is particularly poor for the center-periphery comparison in contrast to pure central or peripheral judgments. In this new experiment, observers compared their perceptual performance for two central stimuli or two peripheral stimuli.

## Experiment 2

### Methods

#### Observers

Eight students from the Justus-Liebig University of Giessen volunteered to take part in the experiment. All volunteers were naïve to the purpose of the experiment, and they had normal or corrected-to-normal visual acuity. Volunteers were reimbursed for their participation. They provided written informed consent in agreement with the Declaration of Helsinki, and all procedures were approved by the local ethics commission (approval number 2017-0030).

#### Apparatus

Same as for Experiment 1, we used the psychtoolbox-3 software (Kleiner, Brainad, & Pelli, 2007) working on MATLAB (http://www.mathworks.com), to display the rendered movies on a linearized Eizo ColorEdge CG245W monitor (10 bits per color channel).

#### Procedure

Again, we used a confidence forced-choice paradigm to investigate metacognitive abilities. In one condition we aimed at replicating Experiment 1 (***center-periphery***condition), while in the other two conditions we investigated metacognitive abilities specific to the central perceptual decisions (***center-center***) and to the peripheral decisions (***periphery-periphery*** condition). To do so, in the ***center-center*** condition we had observers judging the orientation of a central stimulus in both of the intervals, followed by the confidence judgments. We did the same for the ***periphery-periphery*** condition by presenting the stimuli only in the periphery. For each condition, we presented the observers with 500 confidence pairs, resulting in a total of 1,500 confidence trials (3,000 perceptual decisions). We presented our stimuli in 30 blocks of 50 trials from the same condition. The block order was randomized.

#### Analyses & results

As a measure of metacognitive sensitivity, we again resorted to the *confidence* *modulation* *index* (CMI, Equation 1). We first computed perceptual performance in each condition. For each of the ***center-center*** and ***periphery-periphery*** conditions, a single psychometric function was computed because the same stimulus type was presented in both intervals. In contrast, for the ***center-periphery***condition, one stimulus was presented in central vision and the other peripherally. Therefore, for that condition, one psychometric function was computed for the central stimuli and another one for the peripheral ones. CMIs were then computed from these four psychometric functions by splitting the data between confidence-chosen and confidence-declined trials, separately for each participant.

**Figure 4.**
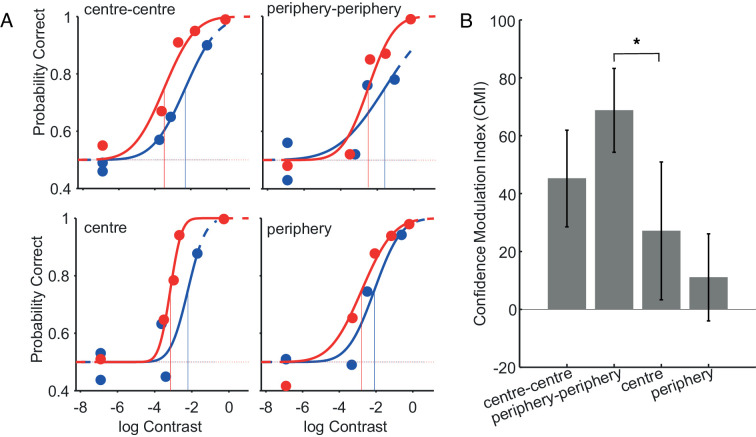
Metacognitive sensitivity for center-center, periphery-periphery, and center-periphery comparisons. A) Perceptual performance for low (blue) and high (red) confidence trials, for the center-center and the periphery-periphery conditions (top left and top right panels, respectively) and for the central and peripheral stimuli in the center-periphery condition (bottom left and bottom right panels, respectively). Contrast on the x-axis, on a log scale. Circles represent data points, continuous lines the fitted psychometric functions. The vertical lines indicate the value on the x-axis for which Φ = 0.5, i.e., the estimated threshold. B) Average CMI (y-axis) for the center-center and the periphery-periphery conditions and for the central and peripheral stimuli in the center-periphery condition. Error bars represent the standard error of the mean.

Figure 4A shows the psychometric functions for one example participant, fitted on the low (red data points and curves) and high confidence trials (blue). High confidence trials always yield lower threshold, indicating metacognitive abilities. The absolute threshold difference is the lowest for the peripheral stimuli in the ***center-periphery*** condition, and likewise, the average CMI (Figure 4B) is also the lowest for that condition. A one way repeated measure ANOVA (with four levels: center-center, periphery-periphery, center, and periphery) indicates significant differences between conditions (*F*(3,21) = 4.77, *p* < 0.05). Post-hoc comparisons shows a significant difference only between the ***periphery-periphery*** condition and the peripheral stimuli in the ***center-periphery*** condition (Figure 4B periphery) (*t*(7) = 5.42, *p* < α; Bonferroni-corrected α = 0.0083), suggesting lower metacognitive sensitivity in the latter condition.

Results from Experiment 2 suggest that the observed metacognitive bias when comparing central and peripheral judgments is associated to relatively poor metacognitive sensitivity. It is possible that when metacognitive sensitivity is low, confidence is at least in part simply based on certain image properties (e.g., eccentricity, size, or contrast) rather than being computed after keeping track of perceptual uncertainty throughout the decision process (Barthelmé & Mamassian, 2010; de Gardelle & Mamassian, 2015).

One visual property that determines perceptual performance in the periphery is stimulus size, and increasing the size of a peripheral stimulus can lead to equivalent discriminability to that found in the fovea (for a review, see Strasburger, Rentschler, & Jüttner, 2011). Observers might report uderconfidence in the periphery because they are aware that peripheral and central stimuli are approximately the same size. Also, observers might have become aware of the higher contrast range that we used to equate peripheral perceptual performance with the central one and used it as a proxy for their confidence: they might have inferred that since they needed higher contrast to perform the task, maybe their performance was lower in the periphery. Such explanations for the underconfidence result may be specific to the stimuli we used and fail to represent a general metacognitive bias. Conversely, if confidence judgments were actually based on performance, it is more likely that the bias generalizes to ecologic situations.

In a third experiment, we tested whether observers based their confidence judgments on contrast or size, resulting in the peripheral underconfidence bias.

## Experiment 3

We tested the hypothesis that observers based confidence judgments on stimulus size or contrast. It is indeed possible that we have knowledge that for stimuli of the same size, peripheral perceptual performance is usually poorer. We should highlight that because we made an effort to match perceptual performance for central and peripheral stimuli in the first two experiments, the contrast used for the peripheral stimuli was indeed higher. If participants did notice the increased contrast of the peripheral stimuli and used this as a cue for confidence, they should display an overconfidence bias for peripheral stimuli. Since we found the opposite bias, it is unlikely that observers mistakenly used perceived contrast as a proxy for confidence. Nonetheless, as a sanity check, we decided to test the effect of stimulus contrast in a third experiment. For this purpose, we increased peripheral and decreased central stimulus size so that lower peripheral contrast was now sufficient to match central performance. The fact that peripheral perceptual performance can reach central performance by increasing relative peripheral stimulus size is usually attributed to cortical magnification (for a review, see Strasburger et al., 2011).

### Methods

#### Observers

Ten students from the Justus-Liebig University of Giessen volunteered to take part in the experiment. All volunteers were naïve to the purpose of the experiment, and they had normal or corrected-to-normal visual acuity. Volunteers were reimbursed for their participation. They provided written informed consent in agreement with the Declaration of Helsinki, and all procedures were approved by the local ethics commission (approval number 2017-0030).

#### Apparatus

Same as for Experiments 1 & 2.

#### Procedure

**Figure 5.**
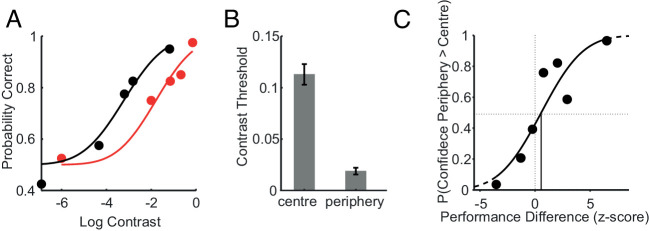
Perceptual performance and metacognitive bias for different sized stimuli. A) Psychometric functions for central (red) and peripheral (black) perceptual decisions. The probability of correct answers is plotted as a function of the log contrast of the stimuli. Discs represent binned data and continuous lines represent the fitted psychometric functions. B) Average contrast thresholds for central and peripheral stimuli. The error bars represent the standard error on the mean. Thresholds are now larger for central stimuli than peripheral ones because the former were smaller in size. C) Probability of higher confidence in the periphery as a function of performance difference. Each data point corresponds to a contrast bin (see analysis in Figure 2). The continuous line represents the fitted cumulative Gaussian function. The vertical continuous black line represents the point of equal confidence (PEC).

We repeated Experiment 1 with a few exceptions. The size of the peripheral stimuli was magnified (to 5.5 dva standard deviation) and the size of the central stimuli was reduced (to 0.125 dva standard deviation). The values were chosen after pilot experiments so that higher central contrast was needed to match peripheral performance. We verified this after data collection (Figures 5A, B). We reduced the number of trials to 100, after we verified that the results of Experiment 1 (revealing the overconfidence bias) can be replicated when only the first 100 trials were taken into account.

#### Analyses & results

To verify that we appropriately chose the relative stimulus sizes so that lower peripheral contrast was needed to match central performance (i.e., we adequately compensated for cortical magnification), we fitted psychometric functions to central and peripheral probability of correct orientation judgments. All the functions fitted to central judgments were shifted rightwards with respect to the ones fitted to peripheral judgments (example in Figure 5A), corroborating our choice of relative sizes. This shift is summarized by the higher central thresholds (Figure 5B, *t*(9) = 11.75, *p* < 0.05).

The confidence bias was measured as for Experiment 1. Figure 5C shows the confidence choices of one participant as a function of *performance* *difference*. On average, the point of equal confidence (*PEC*) was significantly larger than zero (mean z-score = 2.16; *t*(9) = 2.51, *p* < 0.05). In other words, higher peripheral *sensory* *evidence* is needed to equate confidence in the center, indicating underconfidence in the periphery.

Results of Experiment 3 replicate those of Experiment 1, and demonstrate that the underconfidence finding reported in Experiment 1 cannot be explained by the strategy of using the stimulus size or contrast as a proxy for confidence.

## Discussion

In a first experiment, we measured metacognitive biases and sensitivity when observers were asked to compare their central and peripheral perceptual performance. Participants needed higher sensory evidence in the periphery to equate confidence probability, indicating underconfidence in the periphery. In a second experiment, we measured metacognitive sensitivity (as expressed by the *confidence* *modulation* *index*[*CMI*]) when observers were comparing their performance for two perceptual decisions at the same location (central or peripheral), and at different locations (central versus peripheral). Metacognitive sensitivity was poorest when observers compared central with peripheral judgments, indicating that the metacognitive deficit we found is specific to the comparison between central and peripheral judgments. In a third experiment, we tested the hypothesis that observers based confidence judgments on stimulus size or contrast rather than on perceptual performance. Although the relative size of the peripheral stimulus was large enough to require lower peripheral contrast to match central performance, observers again exhibited underconfidence in the periphery.

Our results contribute to a body of experimental evidence that observers trust central vision more than peripheral vision, even when performance is matched and even where it is suboptimal to do so. When asked to indicate the direction of motion of simultaneously presented central and peripheral stimuli, observers are biased towards indicating higher confidence for centrally presented stimuli, even though peripheral and central discrimination performance were equated (Knotts, Lee, & Lau, 2019). Also, when the scotopic foveal scotoma is filled in with surrounding information, observers trust this inferred information in central vision more than veridical information from the periphery (Gloriani & Schutz, 2019). We also showed that humans are impaired in comparing central with peripheral perceptual performance.

Previous work has also found differences in monitoring central and peripheral performance, where the use of peripheral information by the observers was somewhat “inflated,” so as to produce an overconfidence for peripheral vision (Odegaard et al., 2018). In contrast, we found a peripheral underconfidence bias. One possible difference between these two studies is whether perceptual performance is actually matched across all conditions. While we endeavored to match perceptual performance, Odegaard et al. (2018) used an orientation discrimination task where performance was not matched between crowded and uncrowded peripheral stimuli. To overcome this problem, confidence was analyzed after isolating the incorrect trials, resulting in higher confidence for the judgments on crowded rather than noncrowded stimuli, which were thought to be representative of ecological peripheral vision.

We replicated the analysis used by Odegaard et al. (2018) and again found peripheral underconfidence. Specifically, in the data from Experiment 1, we isolated the trials for which the perceptual answer was incorrect, separately for central and peripheral stimuli. Then, for the central incorrect judgments we computed the probability of higher confidence in the center, and for the peripheral incorrect judgments the probability of higher confidence in the periphery. We found that the probability of higher confidence in the center for incorrect central judgments is higher than the probability of higher confidence in the periphery for incorrect peripheral judgments (mean probabilities across participants: 0.5122, 0.2492, for central and peripheral judgments, respectively; *t*(14) = 5.33,*p* < 0.05). Following the logic of Odegaard et al. (2018), this indicates peripheral underconfidence.

Another possible reason for the mismatch between our study and their study is that we directly measure metacognition for the comparison between central and peripheral perceptual performance whereas they compared peripheral uncrowded and crowed stimulation, assuming that the latter one is a representative scenario for ecological peripheral vision. Thus, their results could be specific to crowding and miss the comparison which is representative of the mismatch between our uniform experience of the visual scene and the differences between central and peripheral vision.

In the present study, we did not test peripheral vision under crowded stimulation. We rather preferred to have a clean comparison between central and peripheral vision. However, we think that investigating peripheral metacognition with more ecologically valid stimuli, such us natural scenes, which imply a certain degree of crowding, could potentially add information to our research, as they may include relevant factors that we did not investigate in the current study (e.g., crowding or object boundaries).

Barthelmé and Mamassian (2010) found that observers based confidence judgments on performance, exhibiting no confidence biases for peripheral crowded or uncrowded stimuli. However, their goal was to investigate whether confidence was based on image cues (i.e., contrast) or performance, after dissociating contrast and performance by means of the crowding effect; thus, metacognition for the comparison between central and peripheral perceptual performance was not investigated.

A third possible reason why we did not find inflation is that in our study both the central and the peripheral stimulus were attended. Spatial attention can make detection criterion more conservative, potentially explaining (at least in part) the subjective detailed impression of the entire visual scene, as typically little attention is paid to the periphery (Rahnev et al., 2011). In fact, previous studies reporting inflation have some component of inattention or positional uncertainty involved. For their detection task, Solovey and Colleagues (2015) cued both the central and the peripheral locations, and the stimulus to detect had a 50% chance to show up at either location. Li and Colleagues (2018) explicitly compared perceptual performance between attended and unattended peripheral locations and found more liberal criteria for the unattended locations. In the Odegaard et al. study (2018), spatial attention is potentially impaired because of the positional uncertainty implied by the effect of crowding (Strasburger et al., 2011). We speculate that both perceptual and metacognitive factors may play a role in explaining the perceived uniformity of the visual field. Inflation could play a major role for unattended locations, whereas attention would require filling out peripheral location with information at fixation (Toscani et al, 2017). Our results suggest that we do not experience peripheral vision richer because of a metacognitive bias; we actually know that peripheral vision is poor. The perceived richness of peripheral vision and uniformity of the visual scene is probably due to perceptual mechanisms, at least for attended peripheral regions. Trans-saccadic integration helps to build a uniform visual scene by integrating the information from the same spatial position but different retinal locations (e.g., Ganmor, Landy, & Simoncelli, 2015; Wolf & Schütz, 2015; Herwig & Schneider, 2014; Herwig, Weiß, & Schneider, 2015). This mechanism may add the richness of central vision to peripheral vision only to the locations which are seen peripherally and centrally in two different moments, typically when we direct a saccade to something we first saw in our peripheral vision. In natural vision we do not systematically sample the full visual scene; rather, we focus on task relevant elements and keep the rest in peripheral vision (Hayhoe, 2000; Hayhoe, Shrivastava, Mruczek, & Pelz, 2003; Schütz, Braun, & Gegenfurtner, 2011). In these circumstances other mechanisms could yield the richness of peripheral vision by adding the missing details. Postsaccadic foveal feedback recalibrates the perception of size between the center and periphery (Bosco, Lappe, & Fattori, 2015; Valsecchi & Gegenfurtner, 2016). This recalibration mechanism probably happens because the visual system learns the contingency between peripheral stimulation and how this stimulation would be viewed centrally. Another mechanism is to fill out peripheral vision with perceptual information from central vision. There is evidence that the visual system propagates the brightness at fixation to influence the brightness of areas in the periphery (Toscani et al., 2017). Crucially, this mechanism is selectively applied within an object's boundary, where it is reasonable to assume a certain continuity between central and peripheral brightness. Observers tend to fixate the objects at locations that yield the most diagnostic information when estimating lightness and brightness (Toscani, Valsecchi, & Gegenfurtner, 2013a, 2013b; Toscani, Zdravkovic, & Gegenfurtner, 2016), so it makes sense that the information gathered at the fixated location is filled out to the less diagnostic locations.

However, our results do not exclude that metacognition plays a role in not noticing the difference between central and peripheral vision, as evidence suggests that we perform relatively poorly when we compare our central and peripheral perceptual performance.

Remarkably, our experiment shows that perception and metaperception are not the same. While it is true that our visual system can trick us into perceiving the world as uniform, probably to avoid distraction and surprise whenever we move our eyes, this does not mean that we trust our perception at face value.
